# Effects of Acute Exercise and Chronic Exercise on the Liver Leptin-AMPK-ACC Signaling Pathway in Rats with Type 2 Diabetes

**DOI:** 10.1155/2013/946432

**Published:** 2013-12-17

**Authors:** Xuejie Yi, Shicheng Cao, Bo Chang, Dalin Zhao, Haining Gao, Yihan Wan, Jiaojiao Shi, Wei Wei, Yifu Guan

**Affiliations:** ^1^Department of Biochemistry and Molecular Biology, College of Basic Medical Sciences, China Medical University, Shenyang 110001, China; ^2^Department of Exercise Science, Shenyang Sport University, Shenyang, China; ^3^Department of Sport Medicine, College of Basic Medical Sciences, China Medical University, Shenyang, China

## Abstract

*Aim*. To investigate the effects of acute and chronic exercise on glucose and lipid metabolism in liver of rats with type 2 diabetes caused by a high fat diet and low dose streptozotocin (STZ). *Methods*. Animals were classified into control (CON), diabetes (DC), diabetic chronic exercise (DCE), and diabetic acute exercise (DAE) groups. *Results*. Compared to CON, the leptin levels in serum and liver and ACC phosphorylation were significantly higher in DC, but the levels of liver leptin receptor, AMPK**α**1/2, AMPK**α**1, and ACC proteins expression and phosphorylation were significantly lower in DC. In addition, the levels of liver glycogen reduced significantly, and the levels of TG and FFA increased significantly in DC compared to CON. Compared to DC, the levels of liver AMPK**α**1/2, AMPK**α**2, AMPK**α**1, and ACC phosphorylation significantly increased in DCE and DAE. However, significant increase of the level of liver leptin receptor and glycogen as well as significant decrease of the level of TG and FFA were observed only in DEC. *Conclusion*. Our study demonstrated that both acute and chronic exercise indirectly activated the leptin-AMPK-ACC signaling pathway and increased insulin sensitivity in the liver of type 2 diabetic rats. However, only chronic and long-term exercise improved glucose and lipid metabolism of the liver.

## 1. Introduction

Leptin deficiency or dysfunction is one of the main causes for insulin resistance (IR) and lipid metabolism disorders [1, 2]. However, patients with type 2 diabetes rarely have a leptin deficiency. It has been found that the majority of type 2 diabetes patients have higher levels of body fat, but normal or increased leptin in the plasma [3–6], indicating leptin resistance (LR). Certain levels of leptin effectively could stimulate AMP-activated protein kinase (AMPK) to phosphorylate acetyl-coA carboxylase (ACC), which in turn reduces the ACC activity, decreases fatty acid synthesis [7], and increases the oxidation of fatty acid (FA) [8], consequently, maintaining the balance of lipid metabolism in the body. Studies have shown that even one week of a high fat diet can cause leptin to increase rapidly, leading to fat accumulation in peripheral tissue IR [9]. Obese persons with high serum leptin levels tend to experience a downregulation of leptin receptor in hypothalamus, adipose tissue, and liver [10], which causes peripheral tissues to become LR and promotes lipid accumulation [11–13]. Excessive lipid deposition in nonfat tissue has been known to have a toxic effect on cells and to reduce sensitivity to insulin, eventually leading to diabetes and metabolic syndrome [14]. Liver plays an important role in the regulation of glucose metabolism and lipid metabolism. Moreover, type 2 diabetes and liver steatosis often coexist [15–17], but the causal mechanism is unclear. The possible trigger for type 2 diabetes might be related to leptin resistance, which further inhibits the liver AMPK-ACC signaling pathway and causes liver and systemic metabolic disorders.

Exercise can reduce body fat by increasing energy consumption and improve the leptin resistance [18, 19]. However, how the leptin resistance is improved remains unclear. Previous studies have shown that exercise could upregulate the expression of leptin receptor and induce changes of the JAK-STAT3 signaling pathway in the hypothalamus and peripheral tissues in leptin-resistant rats [20, 21]. After exercise, normal rats experienced increased skeletal muscle growth, increased activity of the liver AMPK and Malonyl-CoA decarboxylase (MCD) pathways, and decreased activity of ACC, which further increased fatty acid oxidation and reduced glyceride synthesis [22]. Our previous study suggested that chronic and acute exercise could both reduce obesity and decrease blood sugar level in type 2 diabetic rats as well as improve the phosphorylation and gene expression related to improved skeletal muscle; AMPK*α*1/2 and its subunits (AMPK*α*1, AMPK*α*2) reduced ACC phosphorylation [23].

It is unknown, however, whether exercise affects the liver leptin-AMPK signaling pathway or whether it can improve liver lipid metabolism. In our present study, a type 2 diabetic rat model, given a high fat diet and a low dose of STZ, was used to address this question. Acute and chronic exercise variables were chosen to study whether exercise could affect protein expression and protein phosphorylation involved in the liver leptin-AMPK-ACC signaling pathway. The relationship between the liver leptin-AMPK-ACC signaling pathway and lipid metabolism, as well as the effect of acute and chronic exercise on that pathway, was investigated.

## 2. Material and Methods

### 2.1. Animals

15-month-old male SD rats (450–470 g) were provided by the Animal Center of the Academy of Military Medical Sciences of the Chinese People's Liberation Army (certification number SCXK (army) 2007-004). Animals were housed under standard conditions (22 ± 2°C, humidity 50 ± 10%, cycles of 12 h light/12 h dark). Experimental procedures were performed in accordance with the Guidance Suggestions for the Care and Use of Laboratory Animals, formulated by the Ministry of Science and Technology of the People's Republic of China in 1998, and were approved by the Animal Ethics Committee of China Medical University.

### 2.2. Animal Models

Rats were randomly divided into a control diet group (CON) and a high fat diet group (HFD). The control diet contained 57.3% carbohydrates, 18.1% protein, 18.8% cellulose, and 4.5% fat and the high fat diet consisted of 23% soy protein, 19.8% pork fat, 19.8% corn oil, 24.5% sucrose, and 5% cellulose and was supplemented with a 1.4% vitamin mixture, 6.7% mineral mixture, and 0.2% choline bitartrate. After 8 weeks, HFD rats were administered 30 mg/kg STZ (citrated buffer, pH 4.4, Sigma) by intraperitoneal injection, while CON rats were injected with the same volume of citrate buffer (1 mL/kg). Four weeks after injection with STZ, all animals with fasting blood above 7.8 mmol/L and postprandial glucose above 11.1 mmol/L were considered to be diabetic [24, 25]. Diabetic rats were then divided into a diabetic control group (DC), diabetic chronic exercise group (DCE), and diabetic acute exercise (DAE). Body weight, water, and food intake were examined every week.

### 2.3. Chronic Exercise

Rats were trained to swim 10–20 min per session for more than 2 days to reduce water-induced stress. Two or three rats per group were placed in a plastic cylindrical pool of 45 cm in diameter and 60 cm of deep, with a water temperature of 34-35°C. After the initial training, the rats underwent chronic exercise for 1 hour/day, 5 days/week, for 8 weeks. The exercise program was conducted in accordance with the Luciano E program with some modifications [26].

### 2.4. Acute Exercise

Acute exercise was conducted in two 90-minute sessions with a 45-minute interval between each session. The exercise program was performed in accordance with the Chibalin AV program with some modification [27]. Animal model preparation and exercise sessions were conducted in the Shenyang Institute of Physical Education Laboratory.

### 2.5. Blood Samples Collection and Blood Biochemistry

24–36 hours after the final session of chronic exercise or 8–16 hours after acute exercise, all rats were anesthetized by single dose intraperitoneal injection of amobarbital (15 mg/kg). Blood samples were collected from tail veins and serum was separated by centrifugation at 1100 ×g for 10 min. Serum glucose, triglyceride, total cholesterol, free fatty acid, HDL-C, LDL-C, and serum leptin levels were measured using an AutoAnalyzer (RT-1904C; Rayto, China). Serum insulin concentration was determined using a radioimmunoassay described by Laemmli [28].

### 2.6. Insulin Tolerance Test (ITT) and Serum Insulin Quantification

Insulin tolerance tests were performed after blood sample collection. The rats fasted for 12 hours were anesthetized and given 1.5 IU/kg of synthetic insulin (Sigma). Blood samples were collected at 0, 5, 10, 15, 20, 25, and 30 min after injection, centrifuged at 1100 ×g for 15 min at 4°C, and stored at −20°C to determine glucose concentrations. The plasma glucose (*t*_1/2_) was calculated from the slope of the last square fit of the plasma glucose concentration during the linear phase of decline [26]. Serum insulin was also measured.

### 2.7. Liver Sampling

After ITT, the animals were sacrificed under anesthesia by intraperitoneal injection of sodium thiopental (200 mg/kg, following the recommendations of the US National Institutes of Health). The liver was isolated and placed in liquid nitrogen and then immediately transferred to −80°C. Western blotting analysis and the detection of liver glycogen, FAA, and TG were performed later.

### 2.8. Liver Glycogen Content

The frozen livers were weighed, digested with 1 mol/L NaOH (1 : 9 wt/vol) at 80°C for 10 min, neutralized with 1 mol/L HCl, and mixed with 6 mol/L HCl to a final concentration of 2 mol/L HCl. The resulting solution was incubated at 85°C for 2 hours and neutralized again with 5 mol/L NaOH [29]. A glucose hexokinase assay kit (Sigma) was used to determine the concentration of hydrolyzed glucose and glucose content was determined as micromolar per gram of tissue. Liver FFA was determined with fatty acid kit (Sigma) following the manufacturer's instructions. The liver TG was determined with triglyceride determination kit (Sigma) following the manufacturer's instructions [28].

### 2.9. Tissue Extraction and Western Blotting

The frozen liver was thawed, weighed, roughly cut, placed in protein extraction solution (1% Triton X-100, 100 mM Tris, pH 7.4, containing 100 mM sodium pyrophosphate, 100 mM sodium fluoride, 10 mM ethylenediaminetetraacetic acid, 10 mM sodium vanadate, 2 mM phenylmethyl sulfonylfluoride, and 0.1 mg/mL aprotinin), and ultrasonicated at maximum speed at 4°C for 30 s (JY92-IIN; Scientz, Ningbo, China). The homogenate was centrifuged at 9 000 ×g at 4°C for 40 min (HC-3618R; Zonkia, Hefei, China). Nonsoluble material was discarded. The protein concentration in the supernatant was quantified using Bradford's method. Then, 100 *μ*g of tissue extract was mixed with an equal volume of 3× sample buffer solution (6.86 M urea, 4.29% SDS, 300 mM DTT, and 43 mM Tris·HCl, pH 6.8) at room temperature for 30 min, subjected to sodium dodecyl sulfate-polyacrylamide gel electrophoresis (SDS-PAGE, 10% polyacrylamide gels), and transferred to a polyvinylidene difluoride membrane at 4°C for 2 h. The membrane was blocked using trihydroxymethyl aminomethane buffer salt + Tween-20 (TBST) containing 5% bovine serum albumin (Sigma) and washed with TBST (pH 7.4). The antibody was dissolved in TBST containing 1% bovine serum albumin overnight at 4°C. The used antibodies include leptin, leptin receptors, phosphorylated (p)-AMPK*α*1 (Thr^172^), AMPK*α*1, p-AMPK*α*2 (Thr^172^), AMPK*α*2, p-AMPK*α*1/2 (Thr^172^), AMPK*α*1/2, acetyl-CoA carboxylase (ACC), and p-ACC (Ser^79^) (Cell Signaling Technology, Beverly, MA, USA; 1 : 1 000 dilution). Bands of interest were visualized by enhanced chemiluminescence and absorbance was determined using FluorChem V2.0 gel imaging analysis software (Alpha Innotech, San Leandro, CA, USA).

### 2.10. Statistical Analysis

Results were presented as mean ± standard error (SE). Differences between groups were compared by one-way analysis of variance (ANOVA). Values of *P* < 0.05 were considered statistically significant. Statistical analyses were performed using JMP software (SAS Institute, Cary, NC).

## 3. Results

Table 1 reports the body weights and biochemical parameters of different groups of rats. When comparing with the control group, the DC group shows a significant increase of the concentrations of blood glucose, leptin, triglyceride, total cholesterol, free fatty, acid and LDL-C. The DC group also shows the decreased insulin sensitivity and HDL-C level, respectively. On the other hand, the body weight, epididymal fat mass, and blood insulin concentration exhibit small variations. All these changes are the typical metabolism characteristics of type II diabetes, proving that the animal model prepared using high fat diet plus STZ-induced disorders is suitable for the current study.

Data in Table 1 also illustrates the effect of chronic exercise on the DC rats. Chronic exercise could significantly reduce the blood glucose, leptin, triglycerides, total cholesterol, free fatty acids, and LDL-C. In contrast, chronic exercise could also increase insulin sensitivity and HDL-C levels. The acute exercise reduces blood glucose but increases the blood glucose disappearance rate and there were no significant effects on dyslipidemia and plasma leptin concentrations.

Compared to the CON group, liver glycogen of the DC group significantly decreased, but TG and FFA levels were significantly elevated (Table 2). This indicates a disorder of the liver glycolipid reserves and FAA. As shown in Table 2, chronic exercise effectively increased glycogen content and reduced liver TG and FAA, whereas acute exercise seemed to have no effect on the liver glucose and lipid reserves.

**Figure 1:**
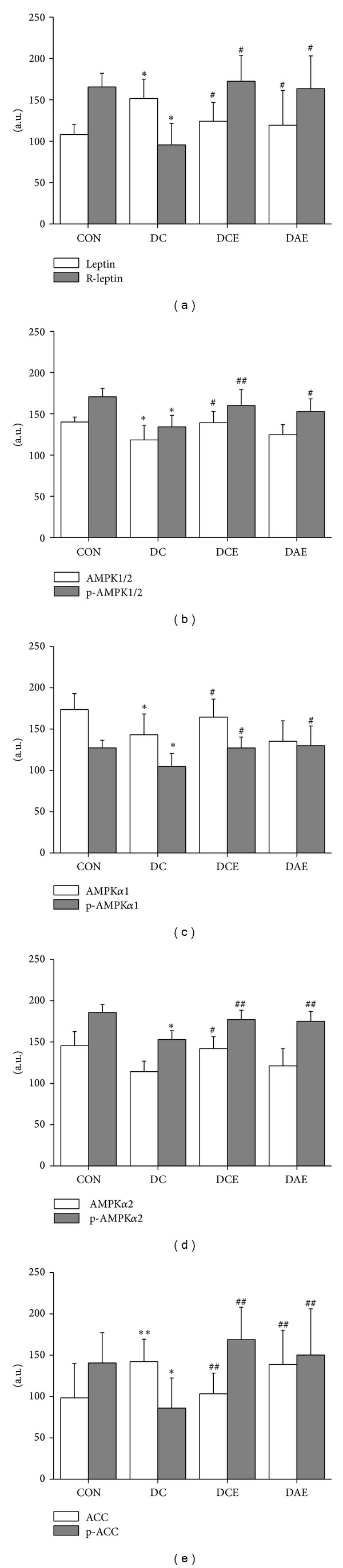
Histograph showing the effects of exercise on the leptin-AMPK-ACC pathway in type 2 diabetes rats. The relative levels of protein phosphorylation and protein expression related to the leptin-AMPK-ACC signaling pathway on different groups were shown. (a) Leptin and its receptor. (b) AMPK1/2 and p-AMPK1/2 (Thr^172^). (c) AMPK*α*1 and p-AMPK*α*1 (Thr^172^). (d) AMPK*α*2 and p-AMPK*α*2 (Thr^172^). (e) ACC and p-ACC (Ser^79^). Note: in comparison with the control group: **P < 0.01, *P < 0.05; in comparison with the diabetes group: ^##^ *P* < 0.01, ^#^ *P* < 0.05 (*n* = 8).

**Figure 2:**
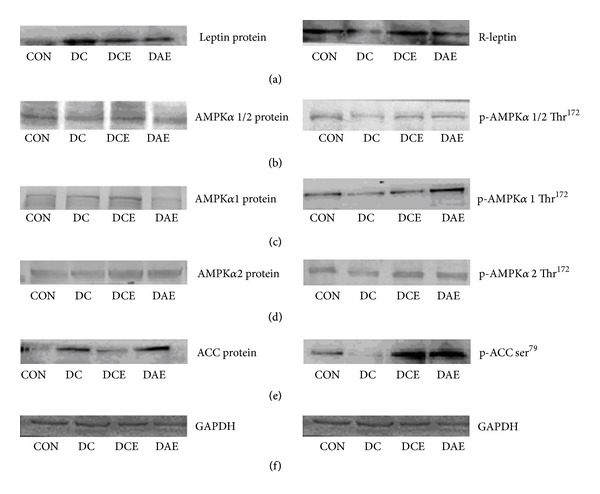
Western blotting images of protein expression and protein phosphorylation related to the leptin-AMPK-ACC signaling pathway of different groups of type 2 diabetes rats. (a) Leptin and its receptor. (b) AMPK*α*1/2 and p-AMPK*α*1/2. (c) AMPK*α*1 and p-AMPK*α*1. (d) AMPK*α*2 and p-AMPK*α*2. (e) ACC and p-ACC. (f) GADPH as an internal standard.

To understand the underlying mechanisms of the different exercises on the type 2 diabetes rats, the protein expression and protein phosphorylation profiles were examined using western blotting (Figure 2) and these image data were quantified with gel analysis software and presented in histograph (Figure 1). The DC group had a significantly increased expression of leptin with respect to that of the control group. The leptin expression returned to the basal level after either chronic or acute exercise (Figure 1(a)). However, the expression of leptin receptor showed an opposite change: a relatively low level of leptin receptor for the DC group and a restored level for the chronic exercise (DCE) and acute exercise (DAE) groups, respectively (Figure 1(a)). Interestingly, the ACC expression exhibited a similar trend to that of leptin, and the phosphorylated ACC exhibited a similar trend to that of leptin receptor (Figure 1(e)).

AMP-activated protein kinase (AMPK) is a kinase responsible for the downstream protein phosphorylation. It is composed of two subunits, AMPK*α*1 and AMPK*α*2. Previous studies have identified several phosphorylation positions on each subunit, and the phosphorylation could occur separately or simultaneously. The most frequently observed phosphorylation position is Thr^172^.

As shown, protein expression and the phosphorylation levels of AMPK*α*1/2, AMPK*α*1, and AMPK*α*2 were greatly reduced in the DC group (Figures 2(b), 2(c), and 2(d)). After chronic exercise, the protein expression and the protein phosphorylation of AMPK*α*1/2, AMPK*α*1, and AMPK*α*2 were elevated back to the normal level in both DCE and DAE groups.

The ACC phosphorylation was reduced in the DC group compared to the control group, which is consistent with the increased liver TG and FFA levels shown in Table 2. It suggests that the disruption of the leptin-AMPK-ACC signaling pathway might be associated with liver lipid deposition. Both chronic and acute exercises could increase the ACC phosphorylation. Chronic exercise can effectively repair the damage of the liver leptin-AMPK-ACC signaling pathway. Therefore, in the DCE group, liver glycogen and the leptin and insulin sensitivity were significantly increased. Moreover, TG and FFA decreased, which helped lowering both blood lipids and blood glucose.

In the DAE group, the leptin-AMPK-ACC signaling pathway was still active 8–16 hours after acute exercise, which increased insulin sensitivity, but had no significant effect on liver glycolipid storage.

## 4. Discussion

Our results indicate that a long-term high fat diet plus low dose of STZ could induce disorders of the whole system and of liver lipids in middle-aged rats, which are associated with both insulin and leptin resistance. These phenomena are similar to the onset, progression, and metabolic characteristics of type 2 diabetes in humans [3–6]. The interruption of the liver leptin-AMPK-ACC signaling pathway might be one of the glucose and lipid metabolism disorders found in type 2 diabetes. Eight weeks of chronic exercise not only effectively improved the leptin-AMPK-ACC signaling pathway, but also alleviated the liver and whole system lipid disorders and partially reversed leptin and insulin resistance. Acute exercise could activate the leptin-AMPK-ACC signaling pathway and reduce the blood glucose level for at least 8–16 hours but has no significant effect on hepatic glucose and lipid metabolism.

It has been widely accepted that excessive fat accumulation is strongly correlated with the insulin resistance and leptin resistance in peripheral tissues (muscle and liver) [14, 30–35]. Excessive nutritional and lipid deposition could lead to an increase in the number of fat cells and further stimulates the leptin secretion. Once leptin binds its receptor, triglyceride synthesis will be inhibited, which further promotes the oxidation of free fatty acids in order to avoid excessive lipid deposition [36] and maintain lean body mass [37]. Some studies have suggested that skeletal muscle leptin can activate the AMPK pathway in two ways. In the first activation, leptin acts directly with skeletal muscle to induce the rapid and transient activation of AMPK, and in the second activation mechanism, one is the *α* adrenal system activating AMPK through the hypothalamus sympathetic skeletal muscle and inducing ACC phosphorylation, which further reduces the synthesis of fatty acids [7]. This activated Malonyl-CoA decarboxylase pathway reduces the level of Malonyl-CoA (MA), inhibits the synthesis of fat, eliminates the inhibition of carnitine palmitoyltransferase 1 (CTP1) [38], promotes long-chain fatty acids in the mitochondrial inner membrane, and increases fat oxidation and decomposition [8, 39, 40]. Lacking leptin or the leptin receptor in diabetic rats (fa/fa and ZDF) reduces the AMPK activity in skeletal muscle and in liver, and promotes fat storage. Administration of leptin or AMPK activators can effectively prevent the development of diabetes [41]. However, the effects on the liver leptin-AMPK-ACC pathway of diabetic rats induced by high fat diet plus low dose of STZ are unclear.

Our results indicate that in the DC group, expression of liver leptin significantly increased, but expression of leptin receptor decreased. Moreover, expression and phosphorylation of AMPK*α*1/2, AMPK*α*1, and AMPK*α*2 were effectively inhibited. The ACC phosphorylation was also inhibited, which was associated with a decline of glycogen and an increase of TG and FFA. This data suggests that the liver leptin-AMPK-ACC signaling pathway is related to hepatic glucose and lipid metabolic disorder and might explain the etiology of fatty liver incidence in patients with diabetes.

Changes in body weight may play a role in the prevention of hyperglycemia caused by regular physical activity, although it is not likely to be the only explanation. In our study, diabetic trained animals showed a slight increase in weight, which puts in doubt whether improvements in insulin sensitivity are consequences of weight gain effects. For this purpose, blood triglyceride, total cholesterol, and free fatty acid concentrations were also examined and found to be remarkably reduced by exercise training. This relative decrease in biochemical parameters in diabetic trained animals may have contributed, at least in part, to their improved insulin sensitivity. Furthermore, diabetic animals were submitted to acute exercise, which had no effect on body weight. While the impact of chronic exercise on leptin was related to physical condition, chronic exercise does not have any effect on the blood leptin levels of athletes but reduces the leptin levels of nonathletes with normal weight [42, 43], obese individuals, and obese rats and also reduces body fat and accumulation of skeletal muscle lipids, which can prevent or mitigate leptin resistance [44]. Appropriate exercise can effectively reduce serum leptin of diabetic rats and humans [45], ease leptin resistance, and inhibit the development of diabetes. The impact of chronic exercise on the leptin receptor is also controversial. Research indicates that chronic exercise could downregulate leptin receptor gene expression in the hypothalamus [46] and improve the insulin resistance of aging rats. Another study suggested that chronic exercise could reduce the expression of the liver leptin receptor gene and decrease plasma leptin levels in rats with a high fat diet [47]. In our previous study, we found that chronic exercise could reduce body fat and blood leptin levels but elevate gene expression of the leptin receptor in the adipose tissue of obese rats as well as improve leptin resistance. In agreement with our previous study, decreased leptin expression and increased expression of the leptin receptor were also found in the high fat diet plus low dose STZ-induced type 2 diabetic rats, 24–36 hours after chronic exercise. In agreement with published data, endurance exercise could also increase gene expression of the hypothalamic leptin receptor and activation of the JAK2-STAT3 signaling pathway, reducing leptin and insulin levels [48]. Increased expression of the leptin receptor was found in hypertrophied triceps of professional tennis players [49].

The impact of chronic exercise on the AMPK pathway was mostly studied in skeletal muscle. Chronic exercise can effectively reduce ceramide levels in the skeletal muscle of the high fat diet rats and restore insulin-stimulated glucose transport in skeletal muscle. Moreover, leptin stimulated phosphorylation in the skeletal muscle AMPK-ACC pathway, and promoted the oxidative decomposition of the FA [44, 50]. In Zucker and OLETF rats, where both lines have metabolic abnormalities, chronic exercise induced increased phosphorylation of ACC, and fat decomposition, as well as reduced fat synthesis in skeletal muscle. The impact of chronic exercise on the liver leptin-AMPK-ACC pathway has not been reported, although increase of liver ACC phosphorylation and AMPK *α*1-*α*2-subunit mRNA/protein expression has been found [51]. In our present study, we found that not only was liver AMPK*α*1/2, AMPK*α*1, and AMPK*α*2 protein expression increased, but increased phosphorylation levels were also seen, which further induced ACC phosphorylation, inhibited TG synthesis, promoted FFA oxidation, reduced lipid storage in the liver, and abolished insulin and leptin resistance. The impact of acute exercise on leptin could be influenced by many factors such as exercise stress and intensity, as well as the physical condition of the individual. Bouassida reported that the effects of one-time exercise on leptin levels were related to energy consumption and exercise time. Energy consumption less than 800 kcal or <60 min of active movement does not change the level of serum leptin; however, energy consumption greater than 800 kcal or ≥60 min could stimulate the lipolysis and reduce serum leptin level [13]. 8–16 hours after acute exercise (90 min × 2), leptin expression was significantly decreased and its receptor expression was significantly increased in the liver of diabetic rats. Moreover, AMPK*α*1/2, AMPK*α*1, AMPK*α*2 and ACC phosphorylation levels increase, but no significant effects are noticeable on liver lipid and glycogen storage. Our data suggested that acute exercise could activate the liver leptin-AMPK-ACC signaling pathway, promote lipid mobilization, and inhibit lipid synthesis. This result has also been shown in other tissues. In agreement with our previous studies, AMPK*α*Thr^172^ and the ACC phosphorylation levels were increased in skeletal muscle after 3 hours of acute exercise [23]. Enhanced skeletal muscle AMPK*α*2 activity, AMPK*α*2, and ACC phosphorylation levels were also found in similar human studies [52, 53]. Moreover, Koh et al. further confirmed that acute treadmill exercise reduces ACC adipocyte activity in rats [54].

## 5. Summary

Impaired liver leptin-AMPK-ACC signaling pathways were closely related to glucose and lipid metabolism disorders in high fat diet plus low dose of STZ-induced type 2 diabetic rats. We further confirmed that chronic exercise could indirectly repair the leptin-AMPK-ACC signaling pathway in these rats, alleviate liver and body lipid disorders, and improve the IR and LR. Acute exercise could also indirectly activate the liver leptin-AMPK-ACC signaling pathway and increase insulin sensitivity, but it should be noted that irreversible liver lipid disorders are induced by a high fat diet.

## Figures and Tables

**Table 1:** Body weight and biochemical parameters of different groups.

	CON	DC	DCE	DAE
Weight (g)	520.7 ± 12.00	502.8 ± 10.51	527.3 ± 10.77	507.3 ± 13.16
Epididymal fat (g)	8.83 ± 0.43	7.86 ± 0.34	8.61 ± 0.44	8.12 ± 0.45
Insulin (pmol/L)	66.14 ± 2.80	76.29 ± 4.17	70.86 ± 3.61	68.70 ± 3.34
FBG (mmol/L)	5.35 ± 0.23	13.18 ± 0.52**	11.57 ± 0.42^#^	11.51 ± 0.41^#^
Kitt (%/min)	3.19 ± 0.19	1.95 ± 0.30**	2.87 ± 0.22^#^	2.61 ± 0.15^#^
TG (mmol/L)	1.68 ± 0.09	2.06 ± 0.11**	1.79 ± 0.10^#^	2.12 ± 0.07^$^
TC (mmol/L)	4.15 ± 0.26	5.78 ± 0.39**	4.69 ± 0.21^#^	5.67 ± 0.35^$^
FFA (mmol/L)	0.39 ± 0.01	0.63 ± 0.05**	0.49 ± 0.04^#^	0.60 ± 0.04
HDL-C (mmol/L)	1.730 ± 0.33	1.029 ± 0.10**	1.358 ± 0.16^#^	0.94 ± 0.29
LDL-C (mmol/L)	0.27 ± 0.12	1.21 ± 0.24**	0.51 ± 0.19^##^	1.25 ± 0.35^$$^
Leptin (ng/mL)	3.25 ± 0.38	5.39 ± 0.64**	4.41 ± 0.61^#^	5.28 ± 0.49

Results were expressed as mean ± standard error (*n* = 7-8). Differences between groups were compared by one-way analysis of variance (ANOVA).

Note: in comparison with the control group: **P < 0.01, *P < 0.05; in comparison with the diabetes group: ^##^ *P* < 0.01, ^#^ *P* < 0.05; in comparison with the chronic exercise group: ^$$^ *P* < 0.01, ^$^ *P* < 0.05.

**Table 2:** The effects of exercise on liver glycolipid storage in type 2 diabetes rats.

	CON	DC	DCE	DAE
Hepatic G (mg/g)	42.31 ± 5.80	19.86 ± 2.32*	38.45 ± 6.34^#^	17.64 ± 4.67
Hepatic TG (*μ*mol/g)	35.2 ± 20.1	144.1 ± 50.4**	51.0 ± 15.3^##^	138.4 ± 33.4
Hepatic FAA (*μ*mol/g)	103.4 ± 35.8	179.4 ± 36.2**	116.9 ± 27.5^#^	163.9 ± 43.9

Results were expressed as mean ± standard error (*n* = 7-8). Differences between the groups were compared by one-way analysis of variance (ANOVA).

Note: compared with normal control group: **P < 0.01, *P < 0.05; compared with the diabetes control group: ^##^ *P* < 0.01, ^#^ *P* < 0.05.
